# Evaluation of Novel Combined CBC‐Derived Systemic Inflammatory Ratios and Their Dynamic Changes as ICU Mortality Predictors, a Retrospective Cohort

**DOI:** 10.1002/hsr2.70441

**Published:** 2025-03-05

**Authors:** Helia Azmakan, Farshad Hashemian, Kaveh Kazemian

**Affiliations:** ^1^ Department of Clinical Pharmacy, Faculty of Pharmacy Tehran Azad University of Medical Sciences Tehran Iran; ^2^ Toxicology Research Center Aja University of Medical sciences Tehran Iran

**Keywords:** critical illness, inflammatory markers, intensive care unit, lymphocytes, mortality, neutrophils, platelets

## Abstract

**Background and Aims:**

Novel biomarkers, such as neutrophil lymphocyte ratio, monocyte lymphocyte ratio, neutrophil to lymphocyte platelet ratio, derived neutrophil to lymphocyte ratio, systemic immune‐inflammation index, systemic inflammation response index, and aggregate index of systemic inflammation, have shown promising prognostic value, especially in ICU settings. We aimed to evaluate the potential of the mentioned factors as ICU mortality predictors in a heterogeneous ICU cohort.

**Methods and Materials:**

We conducted a retrospective cohort study using data obtained from the intensive care unit (ICU) records of 311 patients. We evaluated the strength of the inflammatory parameters upon admission, 48 h later, and their dynamic changes within this period in predicting ICU mortality. We used multivariate logistic regression with backward elimination, which were further validated using ROC and calibration curves. Interaction terms were added to assess the possible modifications in predictive performance of ratios across various subgroups of patients.

**Results:**

NLPR, 48 h post ICU admission (*p* < 0.001, OR: 7.3436, 95% CI: 3.2986–17.2619) and NLPR changes during the first 48 h of ICU admission (*p* = 0.018, OR: 2.3826, 95% CI: 1.2069–6.7112), were significant predictors of ICU mortality in the multivariate logistic regression models. The model, including 48‐h NLPR, had the highest AUC of ROC, calibration slope, and lowest AIC (0.8671, 0.8622, and 229.12, respectively). Also, the predictive performance of NLPR dynamic changes decreases significantly among patients with a background of CVA.

**Conclusions:**

NLPR level, 48 h post‐ICU admission and its dynamic changes during the first 48 h of ICU stay, significantly predict ICU mortality among heterogeneous critically ill patients. These findings can serve as practical and accessible predictors of ICU mortality, particularly in settings, where traditional scoring systems may not be routinely available.

## 1 Introduction

Systemic inflammation plays a central role in the progression of life‐threatening conditions in critically ill patients [1, 2]. These conditions are commonly observed in the intensive care unit (ICU) and significantly contribute to ICU mortality [3], therefore, early identification of patients at risk of these complications is crucial in directing timely interventions and improving outcomes. Traditionally, ICU severity scoring systems such as APACHE and SOFA have been used to predict outcomes [4, 5]. However, these scores often require complex data inputs and may not be routinely available in all settings. In contrast, inflammatory markers derived from routine blood tests offer a simple, rapid, and cost‐effective method of assessing the body's immune response and inflammatory status.

Recently, several novel inflammatory markers have been identified to diagnose and monitor the progression of various inflammatory and infectious diseases [6, 7, 8, 9]. The monocyte/lymphocyte ratio (MLR), the neutrophil/lymphocyte ratio (NLR), the neutrophil/lymphocyte/platelet ratio (NLPR), the derived neutrophil/lymphocyte ratio (dNLR), the systemic immune inflammation index (SII), the aggregate index of systemic inflammation (AISI), and the systemic inflammation response index (SIRI) are some of these markers. These markers have shown promise in assessing conditions such as cardiovascular pathology [5], ICH (intracerebral hemorrhage) [10], oncology [11], sepsis [12], trauma [13], COPD (chronic obstructive pulmonary disease) [14] and, more recently, in the context of COVID‐19 [15, 16, 17, 18]. Also, a meta‐analysis revealed the importance of NLR in predicting a poor prognosis in patients diagnosed with stroke [19]. These markers provide insight into the balance between the innate immune response, driven by neutrophils, and the adaptive immune response, indicated by lymphocytes [20, 21].

These ratios, especially NLR, have been evaluated and proved to be beneficial in predicting ICU outcomes by several studies. A study by Sara Velazquez et al. evaluates the ability of inflammatory ratios to predict ICU admission among COVID‐19 patients. In this study, neutrophil‐platelet ratio (NPR) was shown to be a significant predictor of ICU admission [22]. Another study evaluated the predictive ability of several combined ratios among CHD (congenital heart disease) patients in the ICU. In this study, as well, inflammatory ratios, including SII, SIRI, NLR, PLR, NLPR, AISI, and RDW, were significantly associated with ICU mortality [23]. Sari et al. showed in their study that NLR is a predictor of ICU mortality and antibiotic responsiveness among patients with sepsis and septic shock [24]. Çakin et al. showed in their study that although NLR, SIRI, CRP/albumin ratio, and MII (multi‐inflammatory indices) proved to be significant 28‐day morality predictors, PIV (pan‐immune inflammation value), SII, PLR (platelet‐lymphocyte ratio), and MLR are not [25]. In a study evaluating NLR, PLR, SIRI, and SII, it was shown that NLR is more accurate in predicting stroke‐associated pneumonia and poor outcomes among ICH patients [10]. Other studies indicated that both low and high levels of SII are significantly associated with hospital and ICU mortality in patients with AKI [26], and also short‐term mortality in critically ill patients with sepsis [27].

The primary aim of this study is to evaluate the prognostic value of inflammatory ratios, including MLR, NLR, NLPR, dNLR, SII, SIRI, and AISI, both as static measurements and dynamic markers reflecting changes during the first 48 h of ICU admission for predicting ICU mortality. Additionally, we aim to establish cut‐off points that are applicable across diverse ICU populations. Based on current literature, there are not enough studies investigating the predictive value of changes in these parameters in the ICU setting, which makes this study among the first to comprehensively evaluate the importance of not only simple and complex inflammatory parameters, but also their changes in predicting ICU mortality among a heterogeneous population, independent of the ICU diagnosis and comorbidities.

## 2 Materials and Methods

### 2.1 Study Design and Data Specifications

**Figure 1:**
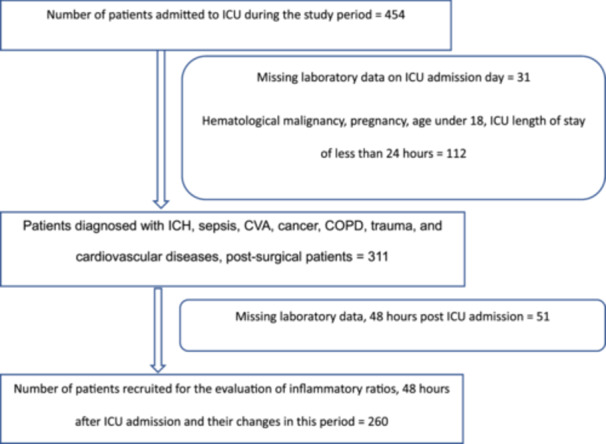
The flow chart for the recruitment of the studied population.

This observational, cohort, retrospective study included patients admitted to the general ICU of a private hospital from September 2020 to November 2021. Patients with missing CBC differential results on their ICU admission day, ICU length of stay of less than 24 h, pregnant women, patients diagnosed with hemotological malignancies, and those who were under 18 were excluded. Patients diagnosed with ICH, sepsis, CVA (cerebrovascular accident), cancer, COPD, trauma, and CVD (cardiovascular disease), as well as postsurgical patients, were included. The patients with these ICU diagnoses were selected due to the availability of sufficient data and supporting evidence from previous literature [5, 10, 11, 12, 13, 14, 19]. The flow chart of the recruitment of the studied population is given in Figure 1.

We gathered data from hospital records into Excel 2019. This data included patient demographics such as age and sex, laboratory results at the time of ICU admission, diagnosis at the time of admission, comorbidities, type of ICU admission (postoperational, emergency room, referral), need for mechanical ventilation, administration of vasopressors, ICU length of stay, and ICU survival as the final outcome.

We categorized comorbidities into 10 categories: CVD, hypertension, diabetes, CKD (chronic kidney disease), COPD, addiction, thyroid disease, CVA, cancer, and neurological diseases.

Laboratory findings, including CBC with differential, sodium, potassium, BUN, and creatinine levels at the time of ICU admission, were recorded. Laboratory data were also documented 48 h after ICU admission, if available, and the change in each parameter during the initial 48 h of the ICU stay was calculated. Additionally, inflammatory ratios including NLR (neutrophil/lymphocyte), MLR (monocyte/lymphocyte), NLPR (neutrophil/(lymphocyte*platelet)), AISI (neutrophil *platelet *monocyte/lymphocyte), SII (neutrophil* platelet/lymphocyte), dNLR (neutrophils/(white blood cells)‐neutrophils), and SIRI (neutrophil* monocyte)/lymphocyte) were calculated.

### 2.2 Statistics

Data were analyzed using SPSS version 28 (SPSS Inc., Chicago, IL, USA) and R version 4.3.3. We used Shapiro–Wilk as normality test, and based on the results, median (Q1‐Q3) or mean ± standard deviation were used to report continuous factors. Categorical variables were reported using frequency and percentage. The association between mortality and categorical variables was determined with either *χ* ^2^ or Fisher's exact test. The sample *t*‐test or Mann–Whitney *U* test was performed for assessment of the association between mortality and continuous variables, where appropriate. The analysis was two‐sided with the significance level set at *p* < 0.05. Receiver Operating Characteristic (ROC) curves and Youden index were used to establish the cut‐off points of numerical parameters for predicting ICU mortality. We acquired multivariate logistic regression models with backward elimination using stepAIC function in R. Two models were conducted for analysis of inflammatory ratios at ICU admission and 48 h post‐ICU admission and one other model was conducted to analyze the changes in the inflammatory indexes within this time span. Missing data in the models were handled through omission. The findings are presented as odds ratios (OR) and 95% confidence intervals (CIs). A *p*‐value under 0.05 was considered significant. The AUCs of the ROC curves of the regression models were obtained to further evaluate the accuracy of the models and they were further validated using calibration curves. Moreover, Akaike information criterion (AIC) of each model was used to compare the predictive performance of the models. Finally, interaction terms were added to the logistic regression models to evaluate the potential modifications in predictive ability across different admission diagnoses and underlying comorbidities.

### 2.3 Ethics

Each patient was assigned an identification code to protect their confidentiality. The study complies with the principles of the Declaration of Helsinki, and approval was appreciated by the institutional ethics committee of Tehran Azad University of Medical Sciences, Tehran, Iran (Ethical Code: IR.IAU.TMU.REC.1400.218). Informed consent was not needed due to the retrospective non‐interventional design of the study.

## 3 Results

### 3.1 Clinical Features

Table 1 provides a concise summary of the demographic and clinical features of the patients. Of the 454 patients, that were admitted to the ICU during the study, 311 met the inclusion criteria. The ICU mortality rate was 27.7% (86/311). Based on ICU outcome, patients were categorized into two groups: survivors (225/311, 72.3%) and non‐survivors (86/311, 27.7%).

**Table 1:** Demographic and clinical findings.

Characteristics ^n^	Survivors^225^	Non survivors^86^	*p*‐value
Age (years)	65 (54–75)	75.5 (61–82.75)	*< 0.001*
Sex (men)^165^	122 (54.2%)	43 (50%)	0.51
comorbidities			
Cardiovascular disease^90^	61 (27.1%)	29 (33.7%)	0.25
Hypertension^164^	125 (55.5%)	39 (45.3%)	0.11
Diabetes^77^	56 (24.8%)	21 (24.4%)	0.93
CKD^45^	27 (12%)	18 (20.9%)	*0.04*
COPD^57^	40 (17.7%)	17 (19.7%)	0.69
Addiction^50^	39 (17.3%)	11 (12.8%)	0.33
Thyroid dysfunction^13^	9 (4%)	4 (4.6%)	0.76^a^
CVA^38^	21 (9.3%)	17 (19.7%)	*0.01*
Cancer^21^	7 (3.1%)	14 (16.2%)	*< 0.001*
Neurologic disorders^12^	9 (4%)	3 (3.5%)	> 0.99^a^
Disability^4^	1 (0.4%)	3 (3.5%)	0.07^a^
Diagnosis			
Surgery^36^	34 (15.1%)	2 (2.3%)	*0.002*
ICH^35^	23 (10.2%)	12 (13.9%)	0.35
Sepsis^36^	23 (10.2%)	13 (15.1%)	0.23
CVA^21^	15 (6.6%)	6 (6.9%)	0.92
Cancer^76^	57 (25.3%)	19 (22%)	0.55
COPD^75^	50 (22.2%)	25 (29%)	0.21
Trauma^15^	11 (4.88%)	4 (4.6%)	> 0.99^a^
Cardiovascular disease^17^	12 (5.3%)	5 (5.8%)	> 0.99^a^
ICU length of stay	10 (6–18)	18 (11–42)	*< 0.001*
Mechanical ventilation^90^	4(1.8%)	86 (100%)	*< 0.001*
Vasopressors^53^	21 (9.3%)	32 (37.2%)	*< 0.001*
Type of ICU admission			
Post operational^114^	98 (43.6%)	16 (18.6%)	*< 0.001*
Emergency room^159^	104 (46.2%)	55 (64%)	*0.005*
Referral^38^	23 (10.2%)	15 (17.4%)	0.08

*Note:* Significant values are written in *italic.*

Abbreviations: CKD, chronic kidney disease; COPD, Chronic obstructive pulmonary disease; CVA, cerebrovascular accident; ICH, intracerebral hemorrhage.

^a^ Fisher's exact test.

Cancer was the most common diagnosis upon admission (24.4%), followed by COPD (24.1%). Most patients were admitted to the ICU from the ER (51.1%) and had underlying cardiovascular disease (65.6%). Also, all deceased patients needed mechanical ventilation and intubation during their ICU stay.

### 3.2 Laboratory Characteristics of the Studied Population

Table 2 represents the median (Q1‐Q3) of inflammatory parameters among deceased patients and survivors. The median of dNLR and NLPR at ICU admission, NLR, NLPR, and dNLR 48 h post‐ICU admission, NLR, NLPR, MLR, AISI, SII, and SIRI changes within the first 48 h of ICU stay were significantly higher among deceased patients, compared to survivors. Moreover, as shown in Table 3, there is no significant association between the levels of inflammatory ratios and ICU admission type in our study.

**Table 2:** Laboratory findings.

At ICU admission ^n^	Survivors^a^	Non survivors^a^	*p*‐value^b^
NLR^311^	8.48 (3.80–16.28)	10.35 (4.67–20.13)	0.08
MLR^311^	0.5 (0.29–0.95)	0.56 (0.27–1.03)	0.85
dNLR^311^	4.32 (2.44–6.69)	5.67 (2.95–8.30)	*0.04*
NLPR^311^	0.046 (0.018–0.094)	0.053 (0.024‐‐0.13)	*0.03*
AISI^311^	684.6 (334.3–2323)	853.61 (376.2–2597.9)	0.98
SII^311^	1492.5 (762.39–3009)	1736.7 (1017.4–4060.9)	0.24
SIRI^311^	4.08 (1.70–10.05)	4.78 (1.86–12.42)	0.72
after 48h^n^	Survivors	Non Survivors	*p*‐value
NLR^260^	6.53 (3.7–10.91)	9.61 (5.5–22.91)	*< 0.001*
MLR^260^	0.45 (0.31–0.74)	0.47 (0.21–0.84)	0.39
dNLR^260^	4.02 (2.14–5.76)	5.13 (3.19–8.54)	*< 0.001*
NLPR^260^	0.033 (0.018–0.064)	0.098 (0.04–0.21)	*< 0.001*
AISI^258^	712.05 (278.51–1425)	614.24 (171.27–2321)	0.60
SII^260^	1287.9 (669.1–2345.8)	1527.6 (463.04–3514.2)	0.78
SIRI^258^	3.56 (1.66‐6.37)	4.34 (1.12–11.44)	0.19
changes in first 48 h^n^	Survivors	Non survivors	*p*‐value
NLR^260^	4.61 (1.72–10.96)	10.20 (2.26–22.02)	*0.007*
MLR^260^	0.26 (0.12–0.55)	0.41 (0.17–1.03)	*0.01*
dNLR^259^	2.32 (0.91‐4.03)	2.66 (0.72–5.46)	0.30
NLPR^260^	0.025 (0.01–0.077)	0.085 (0.017–0.16)	*< 0.001*
AISI^258^	609.88 (178.96–1838.1)	1300.7 (283.44–3308.4)	*0.008*
SII^260^	946.8 (341.73–2618)	1733.1 (550.1–3760.1	*0.02*
SIRI^258^	2.99 (1.09–7.5)	5.83 (1.38–15.25)	*0.01*

*Note:* n: number of patients. Significant values are written in *italics.*

Abbreviations: AISI, aggregate index of systemic inflammation; dNLR, derived NLR; MLR, monocyte to lymphocyte ratio; NLR, neutrophil to lymphocyte ratio; NLPR, neutrophil to lymphocyte platelet ratio; SII, systemic immune‐inflammation index; SIRI, systemic inflammatory response index

^a^ Descriptions are expressed as median (interquartile range).

^b^ Mann–Whitney *U* test.

**Table 3:** frequency of inflammatory ratios' categories across different ICU admission types.

Index^n^	Type of admission: post operation^114^	Type of admission: ER^159^	Type of admission: Referral^38^	*p*‐value
At ICU admission^311^				
NLR > 7.7	67 (39.2%)	85 (49.7%)	19 (11.1%)	0.55
MLR > 0.44	71 (39.7%)	85 (47.5%)	23 (12.8%)	0.32
dNLR > 4.95	55 (37.9%)	75 (51.7%)	15 (10.3%)	0.63
NLPR > 0.055	52 (39.1%)	68 (51.1%)	13 (9.8%)	0.47
AISI > 657	68 (39.5%)	85 (49.4%)	19 (11%)	0.47
SII > 1650	59 (39.3%)	80 (53.3%)	11 (7.3%)	*0.04*
SIRI > 4.67%	26 (42.6%)	30 (49.2%)	5 (8.2%)	0.41
After 48 h^260^				
NLR > 8.3	39 (33.1%)	62 (52.5%)	17 (14.4%)	0.93
MLR > 0.46	44 (34.4%)	72 (56.2%)	12 (9.4%)	0.12
dNLR > 6	23 (30.7%)	42 (56%)	10 (13.3%)	0.90
NLPR > 0.05	41 (35%)	58 (49.6%)	18 (15.4%)	0.45
AISI > 600	50 (34.7%)	78 (54.2%)	16 (11.1%)	0.28
SII > 1690	33 (30.8%)	59 (55.1%)	15 (14%)	0.91
SIRI > 4.55	38 (35.2%)	59 (54.6%)	11 (10.2%)	0.30
Dynamic changes^260^				
NLR > 8.2	28 (26.9%)	60 (57.7%)	16 (15.4%)	0.31
MLR > 0.33	37 (31.1%)	69 (58%)	13 (10.9%)	0.34
dNLR > 3	35 (31.8%)	59 (53.6%)	16 (14.5%)	0.96
NLPR > 0.04	40 (31.7%)	69 (54.8%)	17 (13.5%)	0.96
AISI > 1066	34 (31.2%)	62 (56.9%)	13 (11.9%)	0.63
SII > 1109	39 (30.2%)	74 (57.4%)	16 (12.4%)	0.52
SIRI > 5.5	32 (30.8%)	60 (57.7%)	12 (11.5%)	0.52

*Note:* n: number of patients. Significant values are written in *italics*.

Abbreviations: AISI, aggregate index of systemic inflammation; dNLR, derived NLR; MLR, monocyte to lymphocyte ratio; NLPR, neutrophil to lymphocyte platelet ratio; NLR, neutrophil to lymphocyte ratio; SII, systemic immune‐inflammation index; SIRI, systemic inflammatory response index.

### 3.3 ROC Curves and the Optimal Threshold Points of Inflammatory Indices for Predicting ICU Mortality

We used ROC curves to determine the optimum cut‐off values for inflammatory and hematological parameters. Table 4 displays the results. NLPR after 48 h had the highest sensitivity, specificity, and AUC (0.736, sensitivity = 0.738, specificity = 0.693).

**Table 4:** The area under the curve of inflammatory indexes.

Index	AUC	*p*‐value	95% CI		Cut off point	Sensitivity (%)	1‐Specificity (%)
			Lower	Upper			
At ICU admission							
NLR	0.564	0.08	0.492	0.636	7.7	0.605	0.516
MLR	0.507	0.85	0.432	0.582	0.44	0.616	0.533
dNLR	0.576	*0.04*	0.504	0.648	4.95	0.593	0.418
NLPR	0.579	*0.03*	0.508	0.651	0.055	0.5	0.378
AISI	0.501	0.98	0.477	0.627	657	0.605	0.533
SII	0.543	0.24	0.471	0.616	1650	0.512	0.467
SIRI	0.513	0.72	0.440	0.587	4.67	0.500	0.468
After 48 h							
NLR	0.640	*< 0.001*	0.566	0.714	8.3	0.583	0.392
MLR	0.467	0.39	0.384	0.550	0.46	0.500	0.483
dNLR	0.635	*< 0.001*	0.563	0.708	6	0.440	0.216
NLPR	0.736	*< 0.001*	0.668	0.803	0.05	0.738	0.307
AISI	0.480	0.60	0.396	0.564	600	0.524	0.574
SII	0.511	0.78	0.429	0.593	1690	0.464	0.386
SIRI	0.551	0.19	0.466	0.636	4.55	0.500	0.381
Dynamic changes							
NLR	0.602	*0.007*	0.525	0.678	8.2	0.565	0.313
MLR	0.593	*0.01*	0.519	0.667	0.33	0.571	0.400
dNLR	0.540	0.30	0.462	0.618	3	0.494	0.381
NLPR	0.657	*< 0.001*	0.584	0.730	0.04	0.690	0.369
AISI	0.602	*0.008*	0.510	0.661	1066	0.537	0.371
SII	0.588	*0.02*	0.513	0.664	1109	0.607	0.443
SIRI	0.597	*0.01*	0.521	0.672	5.5	0.524	0.347

*Note:* Significant values are written in *italics*.

Abbreviations: NLR, neutrophil to lymphocyte ratio; MLR, monocyte to lymphocyte ratio; dNLR, derived NLR; NLPR, neutrophil to lymphocyte platelet ratio; AISI, aggregate index of systemic inflammation; SII, systemic immune‐inflammation index; SIRI, systemic inflammatory response index.

### 3.4 Determination of Potential Indicators of ICU Mortality

In univariate analyses, history of CKD, history of CVA, history of cancer, RDW 48 h after ICU admission (cutoff point = 15), BUN on ICU admission (cutoff point = 17.5), Na changes in the first 48 h (cutoff point = 3.5) and age (cutoff point = 75), and administration of vasopressors, along with the inflammatory ratios shown in Table 5, were significantly associated with ICU mortality. They were entered into multivariate logistic regression models with backward elimination and iterative elimination of variables to reach the minimum AIC. The need for mechanical ventilation could not be included in the models due to the fact that all deceased patients in the ICU were mechanically ventilated during their ICU stay. We used distinct multivariate logistic regression models: one including inflammatory ratios on ICU admission day (311 patients), one including the ratios 48 h post‐ICU admission (260 patients), and lastly, one including their changes in this period (260 patients). NLPR and NLR, 48 h post‐ICU admission, were included in separate models to control for confounders. We obtained the ROC curve for each model and calculated the respective AUCs. Also, to extract the calibration slope of the regression models, we used bootstrapping with 1000 samples.

**Table 5:** Univariate and multivariate analysis of factors associated with ICU mortality.

Variables^a^	Univariate analysis		Multivariate analysis	
	*p*‐value	OR (95% CI)	*p*‐value	OR (95% CI)
Age (75 years)	*< 0.001*	3.245 (1.927:5.496)	*< 0.001*	3.5754 (1.756:7.539)
Post operative ICU admission type	*0.006*	0.134 (0.031–0.570)	*0.022*	0.1118 (0.0128:0.6028)
Comorbidities				
CKD	*0.05*	1.941 (0.994:3.724)	—	—
Cancer	*< 0.001*	6.056 (2.421:16.52)	*0.016*	5.079 (5.4239:20.6772)
CVA	*0.01*	2.393 (1.182:4.792)	—	—
Hematological findings				
BUN on admission (17.5)	*0.01*	1.952 (1.173:3.250)	—	—
RDW after 48 h (15)	*0.001*	2.540 (1.483:4.348)	—	—
Na changes during initial 48h (3.5)	*< 0.001*	2.825 (1.64:4.96)	*0.008*	2.6634 (1.3099:5.5825)
Inflammatory indexes				
dNLR on admission (4.95)	*0.006*	2.031 (1.229:3.385)	—	—
NLPR after 48 h (0.05)	*< 0.001*	6.778 (3.817:12.43)	*< 0.001*	7.3436 (3.2986:17.2619)
NLR after 48 h (8.3)	*0.004*	2.171 (1.279:3.384)	—	—
dNLR after 48 h (6)	*< 0.001*	2.859 (1.632:5.009)	—	—
NLR changes in initial 48 h (8.2)	*< 0.001*	3.029 (1.774:5.223)	—	—
MLR changes in initial 48 h (0.33)	*0.01*	1.953 (1.156:3.325)	—	—
NLPR changes in initial 48 h (0.04)	*< 0.001*	3.596 (2.085:6.333)	*0.018*	2.3826 (1.2069:6.7112)
AISI changes in initial 48h (1066)	*0.01*	1.995 (1.173:3.376)	—	—
SII changes in initial 48 h (1109)	*0.01*	1.967 (1.162:3.363)	—	—
SIRI changes in initial 48 h (5.5)	*0.008*	2.06 (1.21:3.52)	—	—

*Note:* Significant values are written in *italics*.

Abbreviations: AISI, aggregate index of systemic inflammation; BUN, blood urea nitrogen; dNLR, derived NLR; Na, sodium; NLR, neutrophil to lymphocyte ratio; MLR, monocyte to lymphocyte ratio; NLPR, neutrophil to lymphocyte platelet ratio; RDW, red cell distribution width; SII, systemic immune‐inflammation index; SIRI, systemic inflammatory response index.

^a^ Cut off values of numerical variables are written in parenthesis.

**Figure 2:**
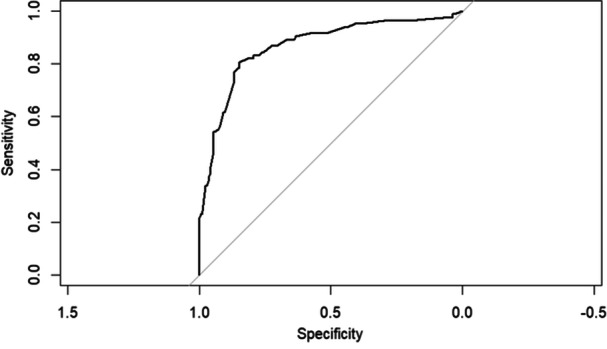
ROC curve of multivariate logistic regression model, including NLPR, 48 h post‐ICU admission. Abbreviation: NLPR, neutrophil to lymphocyte platelet ratio.

**Figure 3:**
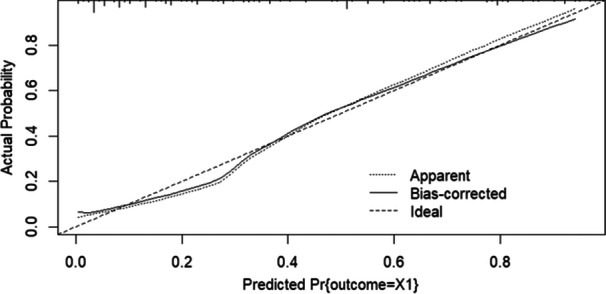
Calibration curve of multivariate logistic regression model, including NLPR, 48 h post‐ICU admission. Abbreviation: NLPR, neutrophil to lymphocyte platelet ratio.

The logistic regression model, which included NLPR 48 h following ICU admission, had the highest AUC of ROC and lowest AIC among the models conducted in this study, suggesting that this model is generally preferred (AUC of ROC = 0.8671, AIC = 229.12). History of CKD, BUN on ICU admission, and dNLR level, 48 h post‐ICU admission, were eliminated from this model. Significant predictors of ICU mortality in this model were age (*p* < 0.001, OR = 3.5754, 95% CI: 1.756–7.539), history of cancer (*p *= 0.016, OR = 5.0795, 95% CI: 1.4139–20.6772), post‐surgery ICU admission (*p *= 0.022, OR = 0.1118, 95% CI: 0.0128–0.6028), and sodium changes in the first 48 h of ICU stay (*p* = 0.008, OR = 2.6807, 95% CI: 1.3099–5.5825), administration of vasopressor (*p* = 0.004, OR = 3.3016, 95% CI: 1.4672–7.6261), NLPR, 48 h post ICU admission (*p* < 0.001, OR = 7.3436, 95% CI: 3.2986:17.2619). This model's calibration slope is 0.8622, indicating excellent calibration with a slight underestimation. The ROC and calibration curves of this model are shown in Figures 2 and 3, respectively. The 10‐fold cross‐validation results of this model also showed good accuracy (AUC of ROC: 0.8472, sensitivity: 0.8713, specificity: 0.7027).

The model, which included NLR 48 h post‐ICU admission, had an AUC of 0.8158, an AIC of 254.57, and a calibration slope of 0.8151. This model showed that NLR, 48 h post‐ICU admission, is not a significant ICU mortality predictor (*p* = 0.46, OR = 1.3678, 95% CI: 0.5885–3.1058). The 10‐fold cross‐validation results of this model are as follows: AUC of ROC: 0.7890, sensitivity: 0.8783, specificity: 0.5513.

The second model included changes in inflammatory ratios in the first 48 h of the ICU stay. The AUC of the ROC of the model was 0.8402. The calibration slope of this model is 0.8251, indicating good calibration with a fair underestimation. The AIC for this model was 241.69. NLPR changes in this model appeared to be a significant predictor of ICU mortality (*p* = 0.018, OR = 2.3826, 95% CI: 1.2069–6.7112). The 10‐fold cross‐validation results of this model indicated acceptable accuracy (AUC of ROC: 0.8106, sensitivity: 0.8709, specificity: 0.5153).

Lastly, the AUC of the model, including the ratios on ICU admissions, was 0.7959. The calibration slope of this model is 0.8059, indicating good calibration with a small underestimation. The AIC of this model was 258.2. In this model, dNLR on ICU admission did not serve as a significant predictor of ICU mortality (*p* = 0.052, OR = 1.8899, 95% CI: 0.9976–3.6304). The 10‐fold cross‐validation results of this model, however, indicated higher sensitivity than specificity (AUC of ROC: 0.7898, sensitivity: 0.8415, specificity: 0.4805).

### 3.5 Modifications of Predictive Ability of Inflammatory Ratios Across Different ICU Diagnoses and Comorbidities

After addition of interaction terms to the relevant logistic regression model, using backward elimination, it was revealed that the predictive significance of NLPR changes becomes weaker in patients with history of CVA (*p* = 0.046, OR = 0.1369, 95% CI: 0.0188–0.9580). However, except for this result, the predictive ability of other inflammatory ratios did not differ significantly in certain subgroups of patients.

## 4 Discussion

This study assessed the predictive ability of various new inflammatory markers, including NLPR, for ICU mortality in a heterogeneous ICU population. Our study indicated that NLPR, assessed 48 h post‐ICU admission, and changes in NLPR during the initial 48 h were significant predictors of ICU mortality.

Studies have demonstrated the central role of the immune system in various complications [6, 28]. Many studies have assessed the combined inflammatory ratios among different cohorts of critically ill patients and suggest that blood lymphocyte and neutrophil‐related indices could serve as potential indicators of mortality [29, 30, 31]. Previous studies claim that NLR and MLR could predict mortality more accurately than monocytes, lymphocytes, and neutrophils separately because they seem to be more unaffected immune pathways in the body [7, 29].

NLPR, when measured 48 h after ICU admission, was the most accurate predictor in our study. It integrates the roles of neutrophils, lymphocytes, and platelets, providing a comprehensive view of the immune‐inflammatory and coagulation processes occurring in critically ill patients. Elevated NLPR values show an increased neutrophil count relative to lymphocytes and platelets. Neutrophils are recognized as innate immune cells that scavenge harmful bacteria and activate other immune cells [20]. Lymphocyte apoptosis is the primary mechanism of lymphocytopenia, which is the main source of immunosuppression [21, 22]. Furthermore, platelets modulate leukocyte activation via serotonin secretion, which not only facilitates the release of cytokines and chemokines but also influences the activation of lymphocytes and neutrophils and regulates other cells' functions, such as monocytes, macrophages, lymphocytes, and endothelial cells [32]. Furthermore, the excessive release of inflammatory mediators and cytokines in critically ill patients leads to disorganized inflammatory responses and disruption of the balance between pro‐inflammatory and anti‐inflammatory mechanisms. This results in an elevation of neutrophils, a reduction in lymphocyte and platelet production, and an increase in their consumption [2]. The inclusion of platelets in NLPR allows it to capture both inflammatory and coagulation pathways, making it a powerful predictor of ICU mortality.

Besides NLPR after 48 h, NLPR changes during the initial 48 h was also a significant predictor of ICU mortality. High NLPR alterations may indicate instability of patient's inflammatory state, which is likely the cause of the higher likelihood of mortality. Very limited studies have evaluated the variations in inflammatory ratios, including NLPR. Only one study evaluated the dynamics of perioperative NLPR for predicting AKI [8].

Su et al. also demonstrated the role of NLPR at admission in long‐term mortality in patients admitted to the ICU due to acute ischemic stroke. This study also indicated that NLPR is a significant predictor of long‐term mortality in these patients, probably due to the reflection of a high inflammation state offered by this factor [33].

Another finding of our study was a significant reduction in the predictive ability of NLPR changes among patients with a history of CVA. However, except for this, the significance of the inflammatory factors did not significantly depend on ICU diagnosis or underlying comorbidities. This shows that the cutoff points obtained in this study apply to different subgroups of patients. This underscores their role as generalizable prognostic tools in the ICU, where rapid and reliable indicators of mortality risk are essential. Moreover, these markers are easily measurable using routine blood tests, making them accessible and cost‐effective tools in the ICU.

There are very limited studies on the investigation of inflammatory ratios in a heterogeneous population. Only one study found that NLR is a significant predictor of hospital mortality among the elderly. This study also found that the predictive ability of NLR decreases in patients suffering from sepsis, pneumonia, and congestive heart failure [34]. Although this study only focused on the elderly and evaluated only one inflammatory index at a single time point.

The main strength of our work is the inclusion of a heterogeneous ICU population and evaluating changes in the ratios. On the other hand, our study has certain limitations, such as the study's retrospective and single‐centered design in a private hospital, which limits the data to what is routinely available as part of standard care. As example, not all patients had complete laboratory data at 48 h post‐ICU admission, either due to discharge or death before this time point or other factors affecting routine data collection. While it reflects real world practices in resource‐limited settings, it may also introduce selection bias, as some patient care practices in this setting may not fully represent other ICU populations. Lack of external validation might also affect the generalizability of our findings. Therefore, future research should aim to validate the generalizability of the findings in larger prospective designs. Finally, certain clinical scores commonly used in ICU settings, such as APACHE and SOFA, were not included in our analysis due to the absence of routine testing of some laboratory tests necessary for calculating these scores (e.g., LFTs and ABGs). These scores are valuable tools for assessment of disease severity in ICU and their absence may limit the ability to directly compare our findings with studies that use them, but it also highlights the potential of inflammatory ratios as practical and accessible markers, especially in resource‐limited settings where such scores are not routinely calculated.

## 5 Conclusion

According to our study, NLPR 48 h after ICU admission and its changes during this time span significantly predict ICU mortality. The predictive performance of the ratios and their cutoff values stayed constant across various diagnoses and underlying diseases, except for the reduction in predictive ability of NLPR changes in patients with a history of CVA. This is a fast, simply available, and cost‐effective factor for prediction of ICU mortality in a diverse critically ill population.

## Author Contributions

**Helia Azmakan:** methodology, writing – original draft, data curation, software, validation, formal analysis, project administration. **Farshad Hashemian:** conceptualization, supervision, writing – review and editing, project administration. **Kaveh Kazemian:** conceptualization, writing – review and editing.

## Conflicts of Interest

The authors declare no conflicts of interest.

## Transparency Statement

The lead author Farshad Hashemian affirms that this manuscript is an honest, accurate, and transparent account of the study being reported; that no important aspects of the study have been omitted; and that any discrepancies from the study as planned (and, if relevant, registered) have been explained.

## Data Availability

The data that support the findings of this study are available from the corresponding author upon reasonable request.

## References

[1] D. C. Angus , W. T. Linde‐Zwirble , J. Lidicker , G. Clermont , J. Carcillo , and M. R. Pinsky , “Epidemiology of Severe Sepsis in the United States: Analysis of Incidence, Outcome, and Associated Costs of Care,” Critical Care Medicine 29, no. 7 (2001): 1303–1310. 11445675 10.1097/00003246-200107000-00002

[2] C. Adrie and M. R. Pinsky , “The Inflammatory Balance in Human Sepsis,” Intensive Care Medicine 26, no. 4 (2000): 364–375. 10872127 10.1007/s001340051169

[3] S. A. Naved , S. Siddiqui , and F. H. Khan , “APACHE‐II Score Correlation With Mortality and Length of Stay in an Intensive Care Unit,” Journal of the College of Physicians and Surgeons—Pakistan 21, no. 1 (2011): 4–8. 21276376

[4] F. L. Ferreira , “Serial Evaluation of the SOFA Score to Predict Outcome in Critically Ill Patients,” Journal of the American Medical Association 286, no. 14 (2001): 1754–1758. 11594901 10.1001/jama.286.14.1754

[5] E. M. Arbănași , A. V. Mureșan , C. M. Coșarcă , et al., “Neutrophil‐To‐Lymphocyte Ratio and Platelet‐To‐Lymphocyte Ratio Impact on Predicting Outcomes in Patients With Acute Limb Ischemia,” Life 12, no. 6 (2022): 822. 35743853 10.3390/life12060822 PMC9225565

[6] Y. Mae , T. Takata , A. Ida , et al., “Prognostic Value of Neutrophil‐To‐Lymphocyte Ratio and Platelet‐To‐Lymphocyte Ratio for Renal Outcomes in Patients With Rapidly Progressive Glomerulonephritis,” Journal of Clinical Medicine 9, no. 4 (2020): 1128. 32326552 10.3390/jcm9041128 PMC7230792

[7] B. Azab , N. Jaglall , J. P. Atallah , et al., “Neutrophil‐Lymphocyte Ratio as a Predictor of Adverse Outcomes of Acute Pancreatitis,” Pancreatology 11, no. 4 (2011): 445–452. 21968329 10.1159/000331494

[8] Y. Li , Z. Zou , Y. Zhang , et al., “Dynamics in Perioperative Neutrophil‐To‐Lymphocyte* Platelet Ratio as a Predictor of Early Acute Kidney Injury Following Cardiovascular Surgery,” Renal Failure 43, no. 1 (2021): 1012–1019. 34187280 10.1080/0886022X.2021.1937220 PMC8260043

[9] A. Tahavvori , R. Mosaddeghi‐Heris , F. Ghanbari Sevari , et al., “Combined Systemic Inflammatory Indexes as Reflectors of Outcome in Patients With COVID‑19 Infection Admitted to ICU,” Inflammopharmacology 31, no. 5 (2023): 2337–2348. 37550520 10.1007/s10787-023-01308-8

[10] R.‐H. Wang , W.‐X. Wen , Z.‐P. Jiang , et al., “The Clinical Value of Neutrophil‐To‐Lymphocyte Ratio (NLR), Systemic Immune‐Inflammation Index (SII), Platelet‐To‐Lymphocyte Ratio (PLR) and Systemic Inflammation Response Index (SIRI) for Predicting the Occurrence and Severity of Pneumonia in Patients With Intracerebral Hemorrhage,” Frontiers in Immunology 14 (2023): 1115031. 36860868 10.3389/fimmu.2023.1115031 PMC9969881

[11] S. Li , Z. Yang , H. Du , W. Zhang , G. Che , and L. Liu , “Novel Systemic Inflammation Response Index to Predict Prognosis After Thoracoscopic Lung Cancer Surgery: A Propensity Score‐Matching Study,” ANZ Journal of Surgery 89, no. 11 (2019): E507–E513. 31667974 10.1111/ans.15480

[12] D. Wu and H. Qin , “Diagnostic and Prognostic Values of Immunocyte Ratios in Patients With Sepsis in the Intensive Care Unit,” Journal of Infection in Developing Countries 17, no. 10 (2023): 1362–1372. 37956370 10.3855/jidc.17907

[13] Y. Qiu , M. Fitzgerald , and B. Mitra , “Initial Neutrophil and Lymphocyte Ratio as a Predictor of Mortality and ICU Admission After Major Trauma,” Trauma 25, no. 2 (2023): 131–136.

[14] D. Du , G. Zhang , D. Xu , et al., “Association Between Systemic Inflammatory Markers and Chronic Obstructive Pulmonary Disease: A Population‐Based Study,” Heliyon 10, no. 10 (2024): e31524. 38818179 10.1016/j.heliyon.2024.e31524 PMC11137537

[15] M. Seyit , E. Avci , R. Nar , et al., “Neutrophil to Lymphocyte Ratio, Lymphocyte to Monocyte Ratio and Platelet to Lymphocyte Ratio to Predict the Severity of COVID‐19,” American Journal of Emergency Medicine 40 (2021): 110–114. 33309506 10.1016/j.ajem.2020.11.058 PMC7719281

[16] J. Rose , F. Suter , E. Furrer , A. Sendoel , M. Stüssi‐Helbling , and L. C. Huber , “Neutrophile‐To‐Lymphocyte Ratio (NLR) Identifies Patients With Coronavirus Infectious Disease 2019 (COVID‐19) at High Risk for Deterioration and Mortality—A Retrospective, Monocentric Cohort Study,” Diagnostics 12, no. 5 (2022): 1109. 35626265 10.3390/diagnostics12051109 PMC9139590

[17] M. Regolo , M. Vaccaro , A. Sorce , et al., “Neutrophil‐To‐Lymphocyte Ratio (NLR) Is a Promising Predictor of Mortality and Admission to Intensive Care Unit of COVID‐19 Patients,” Journal of Clinical Medicine 11, no. 8 (2022): 2235. 35456328 10.3390/jcm11082235 PMC9027549

[18] E. Moisa , D. Corneci , S. Negoita , et al., “Dynamic Changes of the Neutrophil‐To‐Lymphocyte Ratio, Systemic Inflammation Index, and Derived Neutrophil‐To‐Lymphocyte Ratio Independently Predict Invasive Mechanical Ventilation Need and Death in Critically Ill COVID‐19 Patients,” Biomedicines 9, no. 11 (2021): 1656. 34829883 10.3390/biomedicines9111656 PMC8615772

[19] W. Li , M. Hou , Z. Ding , X. Liu , Y. Shao , and X. Li , “Prognostic Value of Neutrophil‐To‐Lymphocyte Ratio in Stroke: A Systematic Review and Meta‐Analysis,” Frontiers in Neurology 12 (2021): 686983. 34630275 10.3389/fneur.2021.686983 PMC8497704

[20] J. C. Marshall , “Inflammation, Coagulopathy, and the Pathogenesis of Multiple Organ Dysfunction Syndrome,” Critical Care Medicine 29, no. 7 (2001): S99–S106. 11445742 10.1097/00003246-200107001-00032

[21] T. Girardot , T. Rimmelé , F. Venet , and G. Monneret , “Apoptosis‐Induced Lymphopenia in Sepsis and Other Severe Injuries,” Apoptosis 22 (2017): 295–305. 27812767 10.1007/s10495-016-1325-3

[22] S. Velazquez , R. Madurga , J. M. Castellano , et al., “Hemogram‐Derived Ratios as Prognostic Markers of ICU Admission in COVID‐19,” BMC Emergency Medicine 21 (2021): 89. 34315437 10.1186/s12873-021-00480-w PMC8314257

[23] Y. Cheng , Y. Chen , M. Mao , R. Wang , J. Zhu , and Q. He , “Association of Inflammatory Indicators With Intensive Care Unit Mortality in Critically Ill Patients With Coronary Heart Disease,” Frontiers in Immunology 14 (2023): 1295377. 38035097 10.3389/fimmu.2023.1295377 PMC10682191

[24] R. Sari , Z. Karakurt , M. Ay , et al., “Neutrophil to Lymphocyte Ratio as a Predictor of Treatment Response and Mortality in Septic Shock Patients in the Intensive Care Unit,” Turkish Journal of Medical Sciences 49, no. 5 (2019): 1336–1349. 31648506 10.3906/sag-1901-105 PMC7018205

[25] Ö. Çakin , A. Karaveli , M. Yüce Aktepe , A. Gümüş , and Ö. E. Yildirim , “Comparison of Inflammatory Marker Scoring Systems and Conventional Inflammatory Markers in Patients over 65 Years of Age Admitted to the Intensive Care Unit: A Multicenter, Retrospective, Cohort Study,” Journal of Clinical Medicine 13, no. 14 (2024): 4011. 39064051 10.3390/jcm13144011 PMC11277589

[26] L. Jia , C. Li , X. Bi , et al., “Prognostic Value of Systemic Immune‐Inflammation Index Among Critically Ill Patients With Acute Kidney Injury: A Retrospective Cohort Study,” Journal of Clinical Medicine 11, no. 14 (2022): 3978. 35887742 10.3390/jcm11143978 PMC9319546

[27] D. Jiang , T. Bian , Y. Shen , and Z. Huang , “Association Between Admission Systemic Immune‐Inflammation Index and Mortality in Critically Ill Patients With Sepsis: A Retrospective Cohort Study Based on MIMIC‐IV Database,” Clinical and Experimental Medicine 23, no. 7 (2023): 3641–3650. 36930382 10.1007/s10238-023-01029-w PMC10022570

[28] A. Bagherimoghaddam , H. Rafatpanah , and H. Mansouritorghabeh , “Elevated Levels of C3, C4, and CH50 of the Complement System in ICU and Non‐ICU Patients With COVID‐19,” Health Science Reports 5, no. 2 (2022): e519. 35224220 10.1002/hsr2.519 PMC8850208

[29] L. Venkatraghavan , T. P. Tan , J. Mehta , A. Arekapudi , A. Govindarajulu , and E. Siu , “Neutrophil Lymphocyte Ratio as a Predictor of Systemic Inflammation‐A Cross‐Sectional Study in a Pre‐Admission Setting,” F1000Research 4 (2015): 123. 26213612 10.12688/f1000research.6474.1 PMC4505778

[30] Y. Shi , C. Yang , L. Chen , M. Cheng , and W. Xie , “Predictive Value of Neutrophil‐To‐Lymphocyte and Platelet Ratio in In‐Hospital Mortality in Septic Patients,” Heliyon 8, no. 11 (2022): e11498. 36439769 10.1016/j.heliyon.2022.e11498 PMC9681647

[31] I. D. Botoș , C. Pantiș , C. Bodolea , et al., “The Dynamics of the Neutrophil‐To‐Lymphocyte and Platelet‐To‐Lymphocyte Ratios Predict Progression to Septic Shock and Death in Patients With Prolonged Intensive Care Unit Stay,” Medicina 59, no. 1 (2022): 32. 36676656 10.3390/medicina59010032 PMC9861709

[32] M. Mauler , C. Bode , and D. Duerschmied , “Platelet Serotonin Modulates Immune Functions,” Hämostaseologie 36, no. 01 (2016): 11–16. 25693763 10.5482/HAMO-14-11-0073

[33] X. Su , S. Zhao , and N. Zhang , “Admission NLPR Predicts Long‐Term Mortality in Patients With Acute Ischemic Stroke: A Retrospective Analysis of the MIMIC‐III Database,” PLoS One 18, no. 8 (2023): e0283356. 37616313 10.1371/journal.pone.0283356 PMC10449205

[34] M. Di Rosa , J. Sabbatinelli , L. Soraci , et al., “Neutrophil‐To‐Lymphocyte Ratio (NLR) Predicts Mortality in Hospitalized Geriatric Patients Independent of the Admission Diagnosis: A Multicenter Prospective Cohort Study,” Journal of Translational Medicine 21, no. 1 (2023): 835. 37990223 10.1186/s12967-023-04717-z PMC10664513

